# Insights on the Evolutionary History and Genetic Patterns of *Octopus vulgaris* Cuvier, 1797 in the Northeastern Atlantic Using Mitochondrial DNA

**DOI:** 10.3390/ani13172708

**Published:** 2023-08-25

**Authors:** Trinidad Pérez, Andrea Romero-Bascones, Negin Pirhadi, Ruth Coya, María del Pino Fernández-Rueda, Isabel Márquez, Lucía García-Flórez, Yaisel J. Borrell

**Affiliations:** 1 Department of Functional Biology, Genetics, University of Oviedo, 33006 Oviedo, Spain; andrearomero095@gmail.com (A.R.-B.); neginprhd@gmail.com (N.P.); coyaruth@uniovi.es (R.C.); borrellyaisel@uniovi.es (Y.J.B.); 2 CEP Fisheries Experimentation Centre, Directorate General of Maritime Fisheries (DGPM), Regional Ministry of Rural Development and Natural Resources from the Principality of Asturias, 33212 Gijón, Spain; mariadelpino.fernandezrueda@asturias.org (M.d.P.F.-R.); lucia.garciaflorez@asturias.org (L.G.-F.); 3 Animal Health Area, SERIDA, 33394 Gijón/Xixón, Spain; imarquez@serida.org

**Keywords:** *Octopus vulgaris*, mitochondrial DNA, *cox1*, control region, D-loop, phylogeography, glacial refugia

## Abstract

**Simple Summary:**

The artisanal octopus fishery is a great tradition and has a large economic impact in Northern Spain. Despite its importance, there is scarce genetic information for this exploited population. The uniqueness of an exploited population and the effects that exploitation may have on its long-term survival is essential information that can be obtained by studying the past and current spatial and temporal genetic patterns. The mitochondrial DNA is used here as a genetic tool to gain insight into Northeast Atlantic octopus populations and their evolutionary history. Although it is not sensitive enough to detect differences in nearby populations, it allows us to differentiate the two main lineages that originated during the Quaternary glaciations. One of the lineages is present exclusively in northern latitudes and the other mainly in the south, although, for the first time, its presence is demonstrated also in the north of the Iberian Peninsula. This implies a more continuous distribution than previously thought for this lineage. We also detect temporal changes in the distribution of the two lineages in contact zones. These changes seem to be associated with the effect of changing oceanographic conditions. Future studies on these associations could be of interest for fisheries in the scenario of global climate change.

**Abstract:**

*Octopus vulgaris* is one of the most harvested octopus species in the world. In the Iberian Peninsula, there are several small-scale fisheries that have a long-term tradition of harvesting octopus. The Asturias fleet (in Northern Spain) has an internationally recognized MSC label for its exploitation. Of concern, genetic assessments of exploited stocks are currently scarce, which could prevent the implementation of adequate managing strategies. We use two mitochondrial regions (cytochrome oxidase subunit 1 and control region) to analyze the genetic status and evolutionary events that conditioned octopus populations’ characteristics in the Northeastern Atlantic. A total of 90 individuals were sampled from three different localities in the Iberian Peninsula as well as a location in Macaronesia. Temporal genetic analyses on Asturias and Algarve populations were also performed. Results indicated the absence of fine spatial genetic structuring but showed the Canary Islands (in Macaronesia) as the most distinct population. Our analyses detected two distinct clades, already described in the literature, but, for the first time, we confirmed the presence of the α-southern haplogroup in the Northern Iberian Peninsula. This result indicates a more continuous cline for the distribution of these two haplogroups than previously reported. Temporal changes in the distribution of both haplogroups in contact zones were also detected.

## 1. Introduction

Artisanal fisheries represent a source of employment and economic income for many regions around the world. Overall, 90% of all fishers recorded worldwide belong to this sector [1]. Especially in the Cantabrian Sea, Northern Spain, there are many representative small-scale fisheries with a long-term fishing tradition [2]. Targeted species of these small-scale fisheries are several commercial fish (hake, mackerel, sardine) but also marine invertebrates [2,3]. For instance, the Asturian and Galician fleets in Northern Spain have a long-term tradition involving cephalopods and crustaceans [3]. Despite the economic and social importance of traditional fisheries, they are under vulnerable conditions, mainly due to political or economic interests, the overexploitation of species by industrial fisheries and the effects of environmental changes [4]. Therefore, there is a need for the social and global protection of this sector in different areas to achieve sustainable fishing that allows the survival of artisanal fisheries [5].

One of the main issues hindering the protection of traditional fisheries is a reported lack of biological data about the fish stocks, including the absence of any type of sustainability assessment [6,7]. The problem is even more serious in the case of invertebrates, where the stock situation is often more difficult to evaluate than for other vertebrates due to the lack of adequate models able to capture the biological features of the species [8]. This situation, together with the lack of fisheries’ independent data for some species and/or scientific campaigns that validate these data, highlights the need for an efficient stock evaluation for this marine group [9].

The common octopus, *Octopus vulgaris* Cuvier, 1797, is one of the most commercially valuable species of cephalopods, mainly fished in the Northern Atlantic, the Mediterranean and Western Africa [10]. Spain is one of the countries with the highest demand for octopus and it is also one of the main contributors to European landings of cephalopods [11]. Artisanal octopus fishing with traps in Western Asturias (northwest of Spain) achieved the Marine Stewardship Council’s (MSC) eco-certification in 2016 and was recertificated in 2021. This ecolabel validated the fishery as sustainable and environmentally friendly, since it has a minimal impact on the marine ecosystem [12], making this artisanal fishery the world’s first cephalopod fishery with this accreditation. However, a lack of biological information was then reported by the BUREAU VERITAS IBERIA, which is the certifying body, and they recommended that “information on the knowledge of octopus populations need to be improved” [13]. From this point, several research projects and studies were undertaken around this fishery: octopus stock assessment model development [8], octopus genetic structure assessments using microsatellites [14] and also octopus juvenile survival after catch-and-release experiments, studies on possible bycatch species, such as the knobbed triton (*Charonia lampas* (Linnaeus, 1758)), bait types and socioeconomic studies [15]. These efforts have allowed the recertification of the fishery as sustainable by the MSC recently [15].

DNA analysis using molecular markers is a powerful tool to improve the knowledge of the species dynamics and to help in its sustainable exploitation. From the different molecular markers in use, two of the most frequently adopted are nuclear microsatellites and mitochondrial DNA (mtDNA) [16]. The former allow us to infer precise genetic structuring patterns [17], while mtDNA helps to interpret the history of a species as it gives information about past events. For instance, it can help to clarify how the last glaciation and interglaciation periods affected the distribution of a marine species and identify phylogeographic breaks or barriers [18]. In addition, the distinct rates of mutation of different regions of mtDNA allow us to identify and characterize organisms at the species or population levels [19].

Historically, *O. vulgaris* was considered a globally distributed species. However, during the past twenty years, research based mainly on mtDNA markers [20,21,22,23,24,25] has suggested that populations previously treated as *O. vulgaris* are in fact a species complex of morphologically similar but genetically distinct *vulgaris*-like species. Within this complex, *O. vulgaris* sensu stricto is described as a widely distributed species that occurs from the Northeastern Atlantic down to Midwestern Africa, including the Mediterranean [25,26]. However, some authors argue that there is a substructure within this species in some areas that should be carefully analyzed and may harbor different managing populations [9]. Supporting this, some studies have observed significant genetic structures of *O. vulgaris* within the Mediterranean and between the Atlantic and Mediterranean, suggesting independent management for each basin [27]. The management of the Eastern Atlantic populations is more controversial and still ambiguous. Using mtDNA markers, some studies [28,29] have reported a single-clade compelling population in the Northern Iberian Peninsula and the western coasts of Africa. Meanwhile, others [30,31] observe a clear break between Northern Iberia and Senegal and the presence of two distinct clades, one along the Northeastern Atlantic and another from southern latitudes, both present in Atlantic Macaronesia.

These contrasting findings suggest the need to implement efforts to investigate the genetic units of *O. vulgaris* in the Northeastern Atlantic. This is especially relevant for *O. vulgaris* active fisheries in Northwestern Africa [26,32] and for the long-time harvesters in the Northern Iberian Peninsula [2]. African populations have been previously analyzed in several studies [31,33,34], but we still lack a complete genetic study on exploited octopus populations in the southern area of the Bay of Biscay. Available studies in the Cantabrian Sea are few and only involve nuclear markers [14,35]. In the case of Asturian fisheries, the determination of the management units and the present status of the populations of octopus in the area has been crucial to achieve the objectives demanded for an internationally certified fishery. Moreover, the annual catches and recruitment of this fishery have fluctuated over the years and may have affected population dynamics [8]. This study performed, for the first time, an mtDNA analysis across the Northern Iberian populations as well as a temporal analysis of the harvested populations, something that, until now, has not been previously documented.

The objectives of the present study were (1) to address, for the first time, the presence of spatial and/or temporal genetic structures of *O. vulgaris* within the Northeastern Atlantic using two mitochondrial markers (cytochrome c oxidase subunit I (*cox1*) and control region (CR)) and give insights into whether the mtDNA is a useful tool to implement scientifically based management strategies for the sustainable exploitation of this species; (2) to unravel demographic past events that may have conditioned the current genetic patterns of this valuable species.

## 2. Materials and Methods

**Figure 1:**
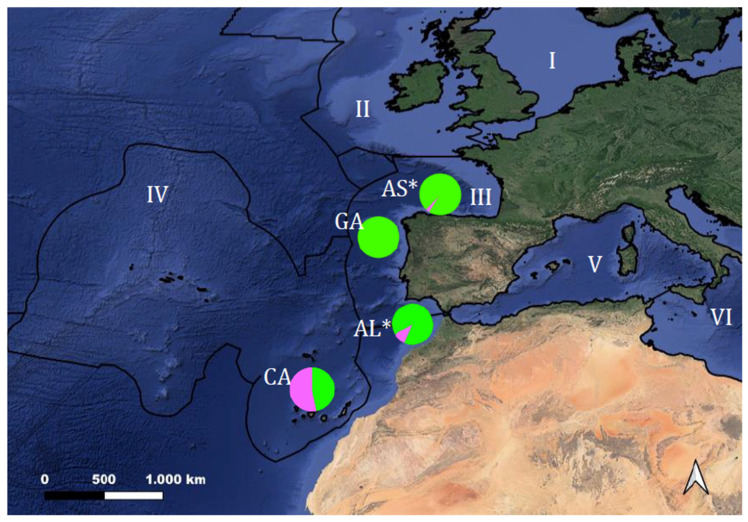
Geographical locations of octopus populations sampled in the present study for genetic analyses of mitochondrial DNA. Sampling sites are AS (Asturias), GA (Galicia), AL (Algarve) and CA (Canary Islands). Colors represents the two clades found for the mitochondrial DNA analysis (in green, haplogroup β, and in pink, haplogroup α, as in Quinteiro et al.’s [30] study). Numbers indicate different marine subregions following the Marine Strategy Framework Directive—MSFD (Directive 2008/56/EC of the European Parliament and of the Council of 17 June 2008, establishing a framework for community action in the field of marine environmental policy): I: North Sea, II: Celtic Sea, III: Bay of Biscay and the Iberian Coast, IV: Macaronesia, V: Western Mediterranean and VI: Ionian Sea. *: Populations sampled in two different fishing seasons. (Information available from the European Environment Agency).

Samples of *Octopus vulgaris* were collected from four different localities in the Northern Atlantic Ocean: two in the Northern Spanish Iberian Peninsula (Asturias, Galicia), one in a continental locality in Algarve, Southern Portugal, and one in the archipelago of the Canary Islands (Macaronesia). All samples (*n =* 15 per site) were collected by artisanal fisheries during the 2020–2021 fishing season, except those from the Canary Islands, which were collected during the 2018–2020 period. Another 30 samples from the 2006–2007 fishing season from two of the previous localities (Asturias and Algarve, 15 samples each) were available, allowing us to test for the existence of genetic changes on a temporal scale (see Table 1 and Figure 1). All 90 samples were collected in tubes with ethanol 70% and stored at 4 °C until needed. Additionally, three samples (one from Basque Country (BC), one from West Mediterranean (VC) and one from East Mediterranean (TK)) were included in some analyses (haplotype networks and phylogenetic trees). DNA was extracted using the E.Z.N.A. Mollusk DNA extraction kit (Omega Bio-tek; Norcross, GA, USA) according to the manufacturer’s instructions and stored at 4 °C until use.

Two mitochondrial loci were analyzed: the cytochrome c oxidase subunit I (*cox1*) and the hypervariable and non-coding mitochondrial control region (CR). Primers used for each locus were OvulCOX11F (5′-TGAATATTYTCAACAAATCAYAAAGAYATTGG-3′) and OvulCOX12R (5′ GGGTGACCAAARAATCAAAATARRTGTTG-3´) for *cox1* and OvulCR3F (5′-GAAAATCTTTCGTGCAAATTACACCACA-3′) and OvulCR4R (5′ TGTTAATGGTCAGGGTCTAAATTCAACTAAAT-3′) for the CR [30]. PCR reactions were carried out following Quinteiro et al. [30], with slight modifications (larger final volume and a fixed MgCl_2_ concentration). They were performed in a final volume of 40 µL containing 2.5 mM of MgCl_2_, 200 μM of dNTP, 0.5 μM of each primer (forward and reverse), 10X Buffer (MgCl_2_ free), 0.5 units of Taq Polymerase (BioTools, Madrid, Spain) and 2.5 µL of a 1:50 DNA dilution. Amplification was carried out in a PE GeneAMP PCR 9700 thermal cycler (Applied Biosystems, Foster City, CA, USA) with an initial step of 3 min at 95 °C followed by 35 cycles (95 °C for 40 s, 60 °C 40 s, 72 °C 40 s) and a final elongation step of 72 °C for 7 min. Products of amplification were checked in a 1% agarose gel and sent to Macrogen Spain for purification and posterior sequencing using the Sanger method (both strands for each amplicon were sequenced). Sequences were analyzed and assembled using Sequencher 4.9 (Gene Codes Corp., Ann Arbor, MI, USA) and manually checked and edited. *Cox1* sequences were checked for species identity in the BOLD database [36,37]. The alignment of the sequences was carried out using the ClustalW program embedded in MEGA X [38].

Basic genetic parameters such as the number of polymorphic sites, haplotype (h) and nucleotide (π) diversity estimates, for each loci separately and for the concatenated sequence, were calculated using DNASP 6.0. [39]. Diverse population parameters such as Tajima´s D and Fu´s F and other genetic differentiation parameters, ϕ_ST_ for population comparisons (10,000 permutations) and AMOVA tests (10,000 permutations), were inferred for the concatenate region in ARLEQUIN 3.5 [40]. One haplotype network was built for the combined dataset using the median joining algorithm in the NETWORK 4 software [41]. The Mantel test was performed to detect isolation by distance with the IBD software v1.52 [42].

We investigated the phylogenetic relationships of these sequences using the sequence of the East Asian common octopus (*Octopus sinensis* d’Orbigny, 1841) as an outgroup (NC052881.1). Neighbor joining (NJ), maximum parsimony (MP), maximum likelihood (ML) or Bayesian approaches were used to construct phylogenetic trees. An NJ tree based on the Jukes–Cantor distance was constructed with MEGA X. The topology of the tree was further investigated by model-free maximum parsimony (MP) as implemented in MEGA X. The MP tree was obtained using the tree-bisection-reconnection algorithm with search level 3, in which the initial trees were obtained with the random addition of sequences (10 replicates). The optimal substitution model for all datasets was estimated using the MODELS tool in MEGA X, where the Hasegawa Kishino Yano (HKY) model [43] showed the highest probability of fitting with the data under analysis. The HKY substitution model was therefore used to obtain a maximum likelihood (ML) tree with the heuristic method of the nearest-neighbor interchange. The reliability of the nodes was assessed by 1000 bootstrap replicates under NJ, MP and ML [44].

To obtain a better representation of the α haplogroup in order to gain better knowledge about the phylogenetic relationships between the two different haplogroups detected, extra data for the CR locus from a previous work by Quinteiro et al. [30] were added. Sequences added to the ones detected in this study represented the most frequent haplotypes, from haplogroup α, in Quinteiro’s work [30]. A haplotype network, based on CR sequences and the full dataset, was built in NETWORK and a Bayesian tree was estimated in MrBayes 3.2 [45]. We executed two independent runs in parallel for each analysis, each consisting of four Metropolis-coupled MCMC chains (one cold, three heated). The tree was performed using 10 million generations, with sampling every 1000 generations, with the first 25% samples discarded as burn-in, and using the HKY model that showed the highest probability of fitting with the data under analysis (lset nst = 2; rates = gamma). Convergence was assessed by the estimated effective sample size (ESS; target value 200). All parameters had ESS values far above the target value (range 6525–7501). The accession numbers for additional haplotypes included in our analyses were MN705218.1, MN705213.1 from Morocco; MN 705258.1, MN705233.1MN 705239.1, MN 705275.1, MN 705266.1, MN705233.1 from Cape Verde; and MN705201.1 from Madeira.

## 3. Results

We amplified and sequenced fragments of *cox1* and CR from 90 individuals (15 per population/fishing season). Three different alignments were created: one for the coding sequence *cox1* of 646 bp length, one for the non-coding sequence CR of 653 bp length (646 bp, indels excluded) and the combined dataset that contained 1299 bp (1292 indels excluded). All samples were unambiguously identified as *O. vulgaris* sensu stricto after *cox1* analysis. The sequences for the different haplotypes detected for CR and *cox1* loci were submitted to GenBank and are available with the submission numbers OR351990-OR352006 and OR346370-OR346378, respectively.

*Cox1* alignment of the 90 individuals resulted in eight different haplotypes defined by 12 variable sites (three of them singletons) and all of them corresponded to synonymous substitutions. The haplotype diversity (h) was 0.659 and the nucleotide diversity (π) was 0.00298. CR displayed a total of 17 different haplotypes defined by 37 polymorphic sites. This region was a non-coding site so higher values of diversity than the coding *cox1* sequence were expected. The global haplotype diversity was h = 0.793 and the nucleotide diversity detected was π = 0.00866. For the concatenated dataset (*cox1* + CR), we found 21 different haplotypes (49 variable sites). Haplotype diversity was h = 0.843 and nucleotide diversity π = 0.00605. All three datasets showed concordant results; the Canary Islands sample had the highest haplotype and nucleotide diversities, and the Algarve 2006–2007 sample showed the lowest values. For the CR and concatenated dataset, the samples from Galicia showed the same haplotype diversity as the Canary Islands samples but lower nucleotide diversity (Table 2).

**Figure 2:**
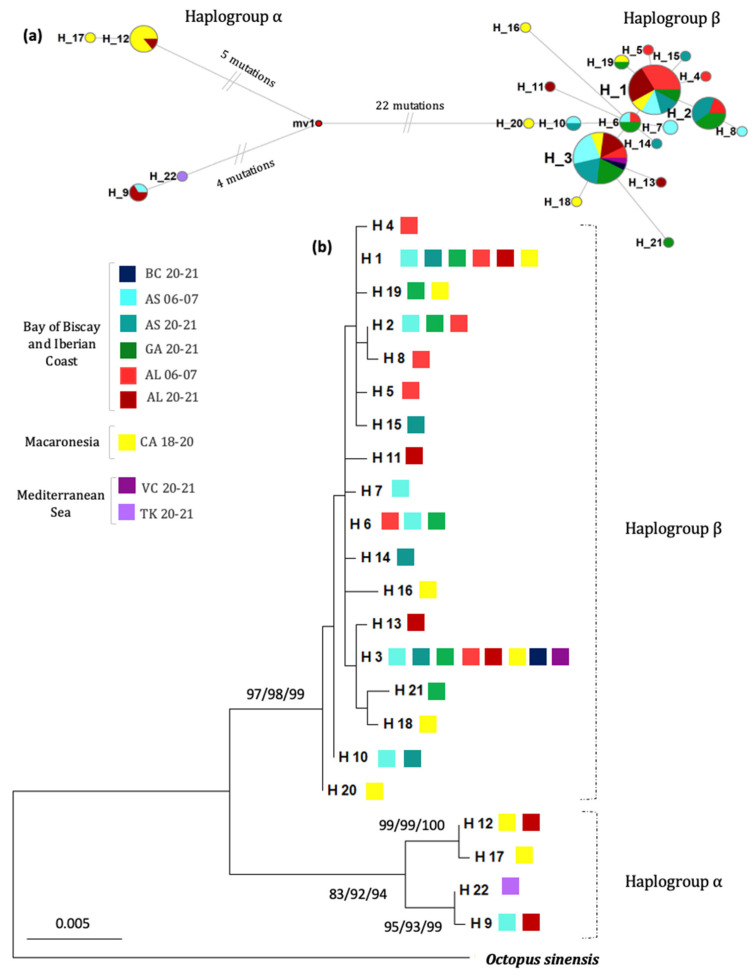
(**a**) Haplotype network built using median joining algorithm for octopus haplotypes (22) from the Northeastern Atlantic using the concatenated mitochondrial region (*cox1* + CR). Circles represent different haplotypes and size is proportional to its frequency. Colors indicate each population and marine subregions are also indicated. The distance for 1 mutation is indicated to be counted from the center of each circle to another. The mv red circles indicate ancestral, extinct or not found haplotypes. (**b**) Maximum likelihood tree for the same haplotypes and samples also using the combined region of *cox1* + CR. Bootstrap support for ML, MP and NJ analysis, respectively, is shown at the nodes (only bootstrap values over 70% are displayed). *Octopus sinensis* is added as an outgroup. The letters in the legend represent the sampling sites (BC (Basque Country), AS (Asturias), GA (Galicia), AL (Algarve), CA (Canary Islands), VC (Valencian Country) and TK (Turkey)). The numbers represent the years in which sampling was performed.

**Figure 3:**
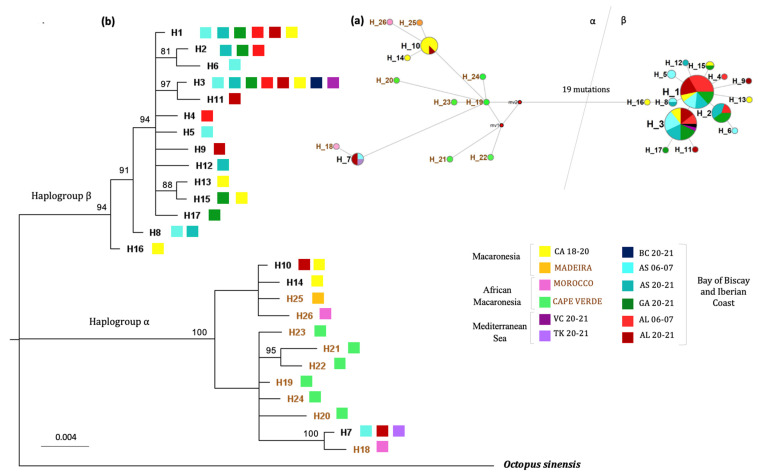
(**a**) Haplotype network for the control region built using median joining algorithm in network for the same octopus haplotypes. Circles represent different haplotypes and size is proportional to its frequency. Colors indicate each population. The distance for 1 mutation is indicated to be counted from the center of each circle to another. Next to haplotypes from Quinteiro et al. [30] are the corresponding numbers in the network. The mv red circles indicate ancestral, extinct or not found haplotypes. (**b**) Bayesian tree inferred in Mr Bayes for the control region of the mitochondria for octopus haplotypes from the Northeastern Atlantic and Mediterranean. It includes individuals from the present study (haplotypes in black) and those from Quinteiro et al. [30] (haplotypes in brown). *Octopus sinsensis* is added as an outgroup. The letters in the legend represent the sampling sites (CA (Canary Islands), VC (Valencian Country) and TK (Turkey), BC (Basque Country), AS (Asturias), GA (Galicia), AL (Algarve)) and the numbers represent the years in which sampling was performed.

The haplotype network built on the concatenated dataset showed two highly divergent haplogroups separated by at least 26 mutations (sequence divergence of 2.01%) (Figure 2a). Haplogroup β was the most common in our samples, representing 87.1% of the individuals studied, whereas haplogroup α showed a lower frequency (12.9%). This haplogroup structure was also observed in the CR sequences (Figure 3a) and, at a lower resolution, the *cox1* sequences (Supplementary Materials, Figure S1). A summary of the sequence differences between haplotypes from the concatenated dataset is available in the Supplementary Materials, Table S1.

A reticulate pattern with several dominant haplotypes showing a star-contraction-like shape was identified for haplogroup β along the Northeastern Atlantic. The two main haplotypes within this group were H1 and H3, present at 25.80% and 27.95 of all individuals (Figure 2a). The parameters for population expansion or neutrality tests (Tajima D and Fu) showed negative values for all the populations when only individuals from this haplogroup were included. Although the majority were not significant, this could suggest a population expansion in this group or clade. The only significant value was the Fu’s Fs statistic for Algarve 2006–2007 (Fu’s Fs = −2.50279; *p* = 0.017). Calculations for the α haplogroup were not performed since this haplogroup was present at a very low proportion or not present at all for the locations examined, except the Canary Islands. Haplogroup α was present in one individual from Asturias 2006–2007, three from Algarve 2020–2021 and eight from the Canary Islands (Macaronesia). Within this group, two small subclades seemed to be present, separated by at least nine mutations (sequence divergence of 0.69%); the first subclade included all the individuals from Macaronesia and one sample from Algarve 2020–2021, and the second subclade included one individual from Asturias 2006–2007, two individuals from Algarve 2020–2021 and the individual from Turkey, Mediterranean (Figure 2a). Remarkably, and for the very first time, an individual from the Northern Iberian Peninsula was clustered in the southern haplogroup α, something not documented before (Figure 2a).

The phylogenetic relationships between the different haplotypes were investigated using the maximum likelihood, maximum parsimony or neighbor joining approaches (Figure 2b). The different methods of tree construction all led to identical topologies, supporting the robust separation of the two main clades. Within group β, there was not any clear clustering or grouping of individuals, with all bootstrap values lower than 70. In contrast, within haplogroup α, the two observed subclades already detected in the haplotype network (one mainly with an individual from Macaronesia and the other including individuals of the Northeastern Atlantic and Mediterranean) were supported by high bootstrap values.

In order to obtain a better understanding of the α haplogroup, a new analysis including more samples belonging to this haplogroup was performed. The new analysis using data obtained by Quinteiro et al. [30] was focused on the control region, because the *cox1* resolution was very low within haplogroup α. Published sequences [30] were from lower latitudinal locations, including Macaronesia (Canarias, Madeira), Morocco and Cape Verde, where this α haplogroup is more frequent.

The new haplotype network (Figure 3a) showed once more the two haplogroups (α and β), separated with at least 20 mutations (sequence divergence of 3.08%). Samples from Cape Verde belonged exclusively to haplogroup α; meanwhile, both haplogroups were present in Macaronesia and the Iberian Peninsula (and Morocco, although no samples from haplogroup β with this origin were included in this analysis). The inclusion of the Cape Verde samples made the differences between haplotypes within haplogroup α more gradual. The number of differences between some Cape Verde haplotypes was similar to the number of differences between individuals of the two subclades previously detected, although they fell somewhere in between both subclades. However, no haplotype was shared between Cape Verde and any of the other locations sampled, either in this study or the one by Quinteiro et al. [30].

Once again, the phylogenetic relationships between the different haplotypes (Figure 3b) supported the separation of the α and β clades and the differences within the α haplogroup. Samples from northern latitudes grouped together with high support and close to Cape Verde samples; in the other group, we had the samples from Macaronesia. Interestingly, samples from Algarve and Morocco could be found in both groups.

Regarding the patterns of genetic differentiation between samples, the ϕ_ST_ values calculated for the concatenated sequence showed that the only population exhibiting significant differences, after Bonferroni correction, was the sample from the Canary Islands 2018–2020 (Table 3). The same result was found when *cox1* and CR sequences were analyzed independently (Supplementary Materials, Tables S2 and S3). The most significant values in all cases were those from the comparison between Canary Islands 2018–2020 and Asturias 2020–2021, Galicia 2020–2021 and Algarve 2006–2007 (Table 3). Although not significant after Bonferroni correction, the differences seen between Algarve 2006–2007 and Algarve 2020–2021 could reflect certain temporal variations within the Algarve locality. Up to three individuals from Algarve 2020–2021 showed an α haplotype; meanwhile, none were detected in the sample from Algarve 2006–2007.

To study the presence of a potential genetic structure within the sampled populations, an AMOVA test was performed using the combined region of both loci (*cox1* + CR). Overall, results revealed global significant genetic differentiation among all the populations studied (Global ϕ_ST_ = 0.21997; *p* = 0.0001). Moreover, the Mantel test revealed a significant positive correlation between the genetic and geographic distance (r = 0.7830, *p* value ≤ 0.0190 by 1000 randomizations). However, there was not a defined population structuring pattern. For instance, clustering groups by aggrupation suggested by pairwise ϕ_ST_ values (Canary Islands population vs. the rest) revealed non-significant differences among groups (ϕ_CT_: 0.43188; *p* = 0.16277), but significant differences were found among populations within groups (ϕ_SC_ =0.02231; *p* = 0.02236). Most of the genetic differentiation was explained between individuals, giving significant differences (ϕ_ST_: 0.44455; *p* = 0.000). When the same analysis was repeated but removing the Algarve 2006–2007 sample (since it had previously shown significant pairwise differentiation with some other samples, although the significance disappeared after Bonferroni correction), no within-groups differences were detected (ϕ_SC_ =0.00864; *p*= 0.13703) and, once again, the ϕ_CT_ values were not significantly different from zero among the groups.

## 4. Discussion

Studying the status of populations is especially relevant in marine species that represent an important social and economic income source and are under intense exploitation by fisheries [6]. In this study, we attempted to assess the genetic patterns (past and present) of populations of *O. vulgaris* within the Northeastern Atlantic using mitochondrial DNA data, including populations that have been scarcely investigated before. The main goal was to gain a better understanding of the origin and distribution of the genetic variability of the species, which can be used to improve the management of the species in this area.

### 4.1. Demography, Phylogenetic and Phylogeography of O. vulgaris Sensu Stricto

Mitochondrial markers are reference markers used to infer the evolutionary history of organisms and give insights into their distribution in the past [19]. Previous works on *O. vulgaris* phylogeography [30,31] have reported two distinct mitochondrial clades along the Northeastern Atlantic with different geographical distributions. Although the two studies did not sample the same localities, they found the presence of two different haplogroups, one in northern areas (Northwestern Iberian coast to Macaronesia) and the other in more southern areas, such as Cape Verde and Senegal. In some cases, an overlap between the two haplogroups was detected in areas such as Macaronesia [30]. Other authors, however, have reported just one haplogroup for all the Eastern Atlantic octopus [29]. Although they detected differences within the haplogroup, they did not recognize it as a different clade. This is probably related to different reasons. For example, their sampling area was wider, including the Western Atlantic and Indian Ocean, and perhaps the variability within each region (i.e., the Northeastern Atlantic) was underestimated due to greater divergence among regions. Most importantly, the use of *cox1* as the only marker [29], which is reported to have a low resolution for the detection of distinct clades, may have been responsible for their subestimation of the groups [30].

In our study, the mitochondrial concatenated region of *cox1* and CR revealed the presence of two distinct clades, as already has been proposed [30,31]. Nevertheless, some interesting differences in the distribution of the two haplogroups were detected in this work. Haplogroup β was again the most frequent along the Northern Iberian coast (87.1% of all individuals analyzed in the present study), with its frequency decreasing in southern latitudes until it completely disappeared in Southern Macaronesia. However, one individual from Asturias (the northern population sampled) was surprisingly clustered within haplogroup α (Figure 1). All the remaining samples within haplogroup α in this study were from southern areas such as Algarve and the Canary Islands, which serve as potential contact areas between both haplogroups. The analysis of the individual from Asturias was repeated twice (starting from DNA extraction) to eliminate genotyping errors, and the same sequence was recovered. In light of this new information, we revisited some previous works and we found that Roura et al. [46], using *cox1* in *O. vulgaris* paralarvae, found one haplotype from Galicia, in the Western Iberian Peninsula, compatible with belonging to the α haplogroup. However, the authors did not highlight this fact, probably for different reasons, the first and most important being the low resolution of *cox1* in differentiating both haplogroups and the second being the low number of samples that could potentially belong to the α haplogroup; the third was that mainly samples from Galicia and, to a lesser extent, from Morocco were analyzed, and, lastly, the authors were focused on other types of questions. Therefore, this is the first time that the presence of this haplogroup α has been confirmed in northern latitudes using *cox1* and CR markers, leading to more questions about the evolutionary theory of the origin of both groups. According to Quinteiro et al. [30], haplogroup α originated in an ancestral refugia on the northwestern coast of Africa, when glaciated periods forced organisms to move towards southern latitudes. Haplogroup β, on the other hand, probably originated from the evolution of variants separated in glaciation periods, showing recent population expansion under favorable conditions.

In this work, the higher genetic variation (especially nucleotide diversity) found in populations within clade α matched with the hypothesis of glaciation refugia suggested by Quinteiro et al. [30]. However, the finding of clade α in samples from the Iberian Peninsula and the Mediterranean, clearly differentiated from those found in areas further south or east, such as Cape Verde, the Azores and Madeira, questioned the hypothesis of a unique glacial refugia in southern latitudes. In fact, previous data indicated the presence of another refugia in the Mediterranean and periods of isolation and subsequent re-connection with the Atlantic [47]. The presence of the same α haplotype in individuals from Asturias, the Azores and the Mediterranean (see H_7 in Figure 3a) supports the idea of these events of secondary contact between the Atlantic and Mediterranean. Whether the presence of these α haplotype individuals in the north of Spain is anecdotal, due to specific weather conditions that allow individuals from southern areas to reach northern latitudes every now and then, or whether there is a small proportion of α haplogroup individuals permanently established in the area, cannot be clarified at this point with just one individual. It would be necessary, therefore, to perform a greater sampling project in this area to elucidate this issue.

For haplogroup β, our data matched with previous studies [30]. Although not significant in our study, populations of this haplogroup all showed negative Tajima D and Fu values, supporting the idea of recent population expansion. Population expansion of one of the lineages is also a common pattern for other northeastern Atlantic marine taxa [48]. The haplotype diversity values for populations within this clade were high, whereas the nucleotide diversity was very low (Table 2), indicating a lack of genetic variation, typical for recolonization areas [49]. On the origin of this clade, it may be that potential small, ancient groups of individuals that found refugia in the north stayed isolated and evolved into new variants, leading to the distinction of both haplogroups. Then, favorable conditions may have allowed the recolonization (along the Western Iberian coast) of this β haplogroup.

### 4.2. Current Genetic Structuring Patterns in the O. vulgaris Northeastern Atlantic Populations

Previous works showed a robust genetic structure within the Northeastern Atlantic associated with the distinct distribution of the two different haplogroups detected [30,31]. Haplogroup α was present in southern locations (i.e., Cape Verde); meanwhile, haplogroup β was present in northeastern sites. In our study, however, such an abrupt cut was not detected. The AMOVA test did not detect any significant structure separating northern and southern latitudes. Instead, our data showed a decline or decrease in the southern clade (α) when moving to northern latitudes and the opposite for haplogroup β (which decreased in frequency from north to south) (Figure 1). These contrasting findings were probably due to sampling differences between works, since previous works based on mtDNA focused on southern populations and just one population from the Northern Iberian coast (Galicia) was sampled. However, our study showed a more continuous sampling range within northern latitudes, which have been poorly studied before. Our results could indicate that the cline or distribution of the haplogroups may have been more continuous than reported in previous studies for the Northeastern Atlantic. Further studies, including more populations in the area, should be carried out to confirm these observations.

Although the absence of fine spatial genetic structuring was observed, we found the Canary Islands population to be significantly different from most of the other populations, with ϕ_ST_ values statistically different from zero. Former studies including the use of microsatellites [14,30,35] have found a genetically distinct group in the Macaronesia region (comprising the Canary Islands, Madeira and the Azores). This finding was also reported in studies with other cephalopods in this region, such as the common cuttlefish (*Sepia officinalis* Linnaeus, 1758) [50]. We found the Canary Islands to have the highest haplotype and nucleotide diversities, associated with the balanced presence of both clades (Table 2). The observed differentiation with respect to other populations of the Peninsula was probably associated with the distance (supported by a significative Mantel test), as reported in previous studies [29], but also to the specific oceanographic conditions, currents and ocean isolation of this geographical area. In fact, the differentiation of *O. vulgaris* in other geographic sites was found to be more related to oceanographic patterns and not necessarily to geographic distance [27,28,47]. The larvae of *O. vulgaris* spend an average 47–54 days in the water current until becoming adults, and even less time in tropical waters, as larvae feed faster and hatch earlier [29]. During this time, they may have limited dispersal, susceptible to oceanographic conditions [51]. The Canaries’ current upwelling region is characterized by the effect of small currents or eddies, which makes it a complex circulatory system that may restrict gene flow or dispersal [52,53]. In addition, the region of Macaronesia is recognized by the European Marine Strategy Framework Directive (MSFD) of the European Commission as a distinct marine subregion (Figure 1), meaning that it harbors specific oceanographic and biogeographical conditions and should then be managed independently. Our findings of genetic differentiation within this area reinforce the distinction of this subregion and support that it should be managed independently.

Finally, we did not find significant differentiation between continental harvested populations (*p* > 0.05) within the Iberian Peninsula. This could be related to the reported higher larvae dispersal in temperate waters, where they spend more time in the water current, which may homogenize populations [29]. However, using microsatellites, Cabranes et al. [35] reported genetic differentiation but only for populations in the Iberian Peninsula located more than 200 km apart. Moreover, Pirhadi et al. [14] did find genetic differentiation between the northern populations (Asturias, Galicia) and the Southern Atlantic area (Algarve). These results show once more how microsatellites are more informative for recent events and may be able to detect more precisely the impact of fishery pressure or other environmental forces on populations than when working with mtDNA [19].

### 4.3. Assessing Temporal Genetic Variation in Exploited Asturian and Algarve O. vulgaris Stocks

Temporal analyses of species under exploitation are another useful tool to evaluate the status of fishing stocks. Most cephalopod harvesters in the Northeastern Atlantic do not regularly assess the status of the populations. This is surprising as octopod species can present highly variable mortality rates and a short life cycle of 1–2 years [9], conditioning the annual abundance of the species that directly depends on the recruitment or successful paralarvae that reach adulthood and enter the population the year before [54,55]. Furthermore, octopus paralarvae are reported to be highly influenced by environmental fluctuations and oceanographic conditions (i.e., changes in sea surface temperature, rainfall and upwelling processes), which may affect the annual variation of the species [55,56]. All these factors necessitate a continuous assessment of the status of octopus populations, especially for artisanal fisheries.

In small-scale fisheries from the Northern Iberian Peninsula, studies on the genetic status of octopus populations are scarce and mainly focused on spatial structure, but Pirhadi et al. [14] recently studied the temporal variation of Asturian populations using microsatellites. The data revealed no significant temporal genetic changes in samples from Olhão (Portugal) within the period of 14 years (approximately nine generations considering *O. vulgaris* life span). No significant temporal differences were found between 2018 and 2021 (two generations) for the rest of the locations, except for the samples from Puerto de Vega (Asturias) collected between 2007 and 2018, with respect to the last sampling collected during 2020–2021 in the same locality [14]. In our study, we did not find significant differences between the two fishing seasons. The haplotype diversity remained the same from 2006–2007 to 2020–2021 (h = 0.819). However, in Asturias 2006–2007, an individual from the ancestral lineage α was found, whereas, in 2020–2021, no haplotype representing this group was present. A larger sampling size may be needed to give insights into whether the ancestral haplotype may have suffered a loss or reduction in Northern Iberia due to intense fishing efforts.

In the Southern Iberian Peninsula, the region of Algarve is reported to be the main Portuguese recruitment region of *O. vulgaris*, where the greatest abundance and representatives of this species are concentrated [57]. Although we did not detect statistically significant differences between seasons for this location, neither in this study using mitochondrial DNA nor in previous studies using microsatellites [14], we detected changes in the distribution of the haplogroups depending on the season. For example, we did not detect any individual from haplogroup α in the 2006–2007 fishing year, in contrast to 2020–2021, where both lineages (α and β) were found. The presence of haplotypes from the α haplogroup in the Algarve 2020–2021 season could be associated with oceanographic conditions (currents, sea surface temperature), which can vary over time and can influence the movement of individuals from certain areas (southern latitudes, Mediterranean) to this area [58]. For instance, the sea surface temperature has shown an increasing trend over the last few years in the region of Algarve [59,60] and it is well known that the paralarvae of *O. vulgaris* are characterized by a preference for warmer temperatures [61]. Regarding the currents, the southern region of Portugal represents a well-known upwelling area. The Portugal current shows a northward direction or southward direction depending on the season and, therefore, could be an important factor in promoting gene flow between Portugal and other, southernmost areas such as the Canarian and North African coasts [62]. The hydrodynamic setting of this area is dominated by the water exchange between the Atlantic Ocean and the Mediterranean Sea (with saltier, warmer and denser water) through the Strait of Gibraltar. The simulated eastward volume flow (Atlantic Ocean to Mediterranean Sea) is generally larger than the westward flow but switches sign during the spring and shows significant interannual variability [63]. The same simulation work showed that a small part of this westward flow can move along the Western Iberian coast and reach the Bay of Biscay [63]. The circulatory pattern of the Gulf of Cádiz’s water masses has traditionally been explained based on the action of prevailing winds in the area: western winds during the winter and eastern component winds for summer (May to September) [55]. It is known that octopus egg laying, in the Gulf of Cadiz, takes place between the spring and summer months and recruitment between October and November [64].

## 5. Conclusions

This study allowed us to estimate the genetic variation and differentiation patterns of populations from the Northeastern Atlantic using mtDNA and it gives insights into the distribution and legacy of the two reported lineages of *O. vulgaris* (α and β) in the past. The use of two mitochondrial markers together proved to be useful to detect the different lineages and to infer the presence of the reported ancestral clade α in northern latitudes, identifying for the first time the presence of this clade in the Iberian Peninsula and supporting the idea of a second glacial refugia in the Mediterranean Sea for this valuable species. However, further studies are needed, increasing the sampling size and number of locations, to further elucidate the evolutionary history of the species.

Regarding the present status of the populations, we did not detect fine spatial genetic structuring using mtDNA but we found differences, probably associated with the distinct distributions of the two clades. The Canary Islands population (showing the greatest frequency of the southern clade among the rest of the sites) was significantly distinct, matching with the hypothesis of isolation by distance and supporting the key role that the oceanographic conditions may have in the restriction of gene flow and the singularity of the Macaronesia area. Nevertheless, the observed lack of differentiation when including all the sampled locations differed from the significant genetic structuring found when studying the same populations but using nuclear markers (microsatellites), suggesting that mitochondrial DNA may not be sensitive enough to update a continuous fishery strategy since it may fail to detect spatially different managing stocks if they are not very distant. Instead, it may be useful to detect temporal changes in the distribution of the two clades in areas of contact—for instance, the Atlantic and Mediterranean (reflected in the frequency of each clade in specific populations)—and associate them with the effect of changing oceanographic conditions or other specific events related to global climate change.

## Figures and Tables

**Table 1:** Number of individuals analyzed in this study (*n*) by fishing season, sampling region and locality. Code, coordinates and FAO fishery division are also included.

Sampling Region	Locality	Code	Coordinates	FAO Division	Fishing Season	*n*
Asturias ^1^ Cantabrian Sea	Puerto de Vega	AS	43.566/−6.633	27.8. c	2006–2007 2020–2021	15 15
Galicia ^1^ Northeastern Atlantic	Bueu	GA	42.363/−8.851	27.9. a	2020–2021	15
Algarve ^1^ Northeastern Atlantic	Olhão	AL	36.947/−7.954	27.9. a	2006–2007 2020–2021	15 15
Canary Islands ^2^ Northeastern Atlantic	San Andrés	CA	28.616/−16.335	34.1.2	2018–2020	15

^1^ Bay of Biscay and the Iberian Coast, ^2^ Macaronesia.

**Table 2:** Population parameters for *cox1* and CR mitochondrial loci and the concatenated sequences from both genes after genetic analyses using octopus samples from the Northeastern Atlantic. Sample size (N), nº of haplotypes (N_h_), sample-specific haplotypes (h_s_), haplotype diversity (h), nucleotide diversity (π), average number of nucleotide differences (k).

		Cytochrome Oxidase 1 (*cox1*)					Control Region (CR)					Concatenated (*cox1* + CR)				
Population	N	N_h_	h_s_	h	π	k	N_h_	h_s_	h	π	k	N_h_	h_s_	h	π	k
Asturias 2006–2007	15	3	0	0.514	0.00168	1.086	6	2	0.790	0.00633	4.095	7	2	0.819	0.00401	5.181
Asturias 2020–2021	15	3	1	0.600	0.00103	0.667	5	1	0.790	0.00179	1.162	6	2	0.819	0.00141	1.829
Galicia 2020–2021	15	3	0	0.600	0.00103	0.667	6	1	0.819	0.00205	1.333	6	1	0.838	0.00154	2.000
Algarve 2006–2007	15	3	1	0.448	0.00074	0.476	4	1	0.552	0.00097	0.629	6	2	0.714	0.00085	1.105
Algarve 2020–2021	15	3	0	0.705	0.00404	2.610	6	2	0.790	0.01330	8.629	6	2	0.790	0.00868	11.238
Canary Islands 2018–2020	15	5	1	0.743	0.00590	3.810	7	3	0.819	0.01966	12.743	8	4	0.838	0.01279	16.552

**Table 3:** Population pairwise ϕ_ST_ values estimated from the mitochondrial concatenated region (*cox1* + CR) in octopus samples from the Northeastern Atlantic. ϕ_ST_ values, using pairwise comparisons as a distance method, are located below the diagonal and above the diagonal with their corresponding *p* values (italics). Significance (* *p* < 0.05). Significance after Bonferroni correction (cut-off value *p* < 0.0033) in bold.

	Asturias 2006–2007	Asturias 2020–2021	Galicia 2020–2021	Algarve 2006–2007	Algarve 2020–2021	Canary Islands 2018–2021
**Asturias 2006–2007**		*0.25304*	*0.34254*	*0.00861*	*0.53163*	*0.00386*
**Asturias 2020–2021**	0.01038		*0.99990*	*0.21156*	*0.11256*	*0.00129*
**Galicia 2020–2021**	0.00265	−0.04978		*0.10068*	*0.10692*	*0.00109*
**Algarve 2006–2007**	0.09957 *	0.03509	0.07656		*0.01346*	*0.00000*
**Algarve 2020–2021**	−0.00602	0.09650	0.09608	0.13592 *		*0.05148*
**Canary Islands 2018–2021**	0.27796 *	0.38857 *	0.38857 *	0.41558 *	0.11231	

## Data Availability

All sequences generated during this study have been deposited in GenBank (https://www.ncbi.nlm.nih.gov/genbank/).

## Supplementary Materials

The following supporting information can be downloaded at: https://www.mdpi.com/article/10.3390/ani13172708/s1, Figure S1: *Cox1* haplotype network; Table S1: Summary of sequences differences between haplotypes for the concatenated dataset; Table S2: Population pairwise ϕ_ST_ values from *cox1* in octopus’ samples; Table S3: Population pairwise ϕ_ST_ values from CR in octopus’ samples.
