# Supplementary material for: Insights on the Evolutionary History and Genetic Patterns of Octopus vulgaris Cuvier, 1797 in the Northeastern Atlantic Using Mitochondrial DNA
Source: Animals (Basel). 2023 Aug 25;13(17):2708. doi: 10.3390/ani13172708 (PMC10486628; doi:10.3390/ani13172708)
Supplement: Supplementary file 1 [file animals-13-02708-s001.zip › animals-2554406-supplementary.pdf]

Figure S1. ***Cox1*** **haplotype network**

built using Median Joining algorithm in Network for the octopus' haplotypes found in the 93 samples analyzed. Circles represent different haplotypes and size is proportional to its frequency. Colors indicate each population.

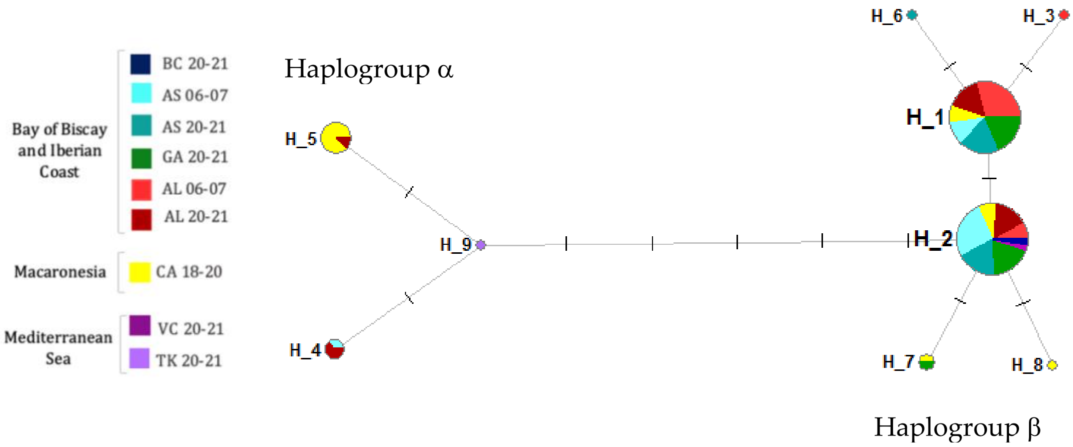

Table S1. Summary of the sequence differences between haplotypes from the concatenated dataset.

The numbers made referent to the position of the variable site within the concatenated sequence. In blue the variable sites from *cox1* sequence and in black the variable sites from the CR sequence. The name of the haplotypes belonging to α haplogroup is depicted in black. The frequency of each haplotype (freq.) is also included in the table.

Haplotype	Variable sites and position within the concatenated sequence	Freq.
	1133344456666667777777777888888899111111111111	
	1149479434445880112467889012577707000000000011112	
	0367283241684089163771190369704723024566888800355	
	64892868918429	
H_1	CTTTCTTACCCCCCTCGTTTATCCTACCACCGTTAGACTTCTGCGCTCT	24
H_2	CTTTCTTACCCCCCTCGTTTATCTTACCACCGTTAGACTTCTGCGCTCT	10
H_3	CTTTCTTACCCTCCTCGTTTATCCTACAACCGTTAGACTTCTGCGCTCT	26
H_4	CTTTCTTACCCCCCTCATTTATCCTACCACCGTTAGACTTCTGCGCTCT	1
H_5	CTTTTTTACCCCCCTCGTTTATCCTACCACCGTTAGACTTCTGCGCTCT	1
H_6	CTTTCTTACCCTCCTCGTTTATCCTACCACCGTTAGACTTCTGCGCTCT	4
H_7	CTTTCTTACCCTCCTCGTTTATCCTACCACCGATAGACTTCTGCGCTCT	2
H_8	CTTTCTTACCCCCCTCGTTTACCTTACCACCGTTAGACTTCTGCGCTCT	1
**H_9**	TCATCCTATTCTCACCGCCTGTTCTGTAGTCATCAAACCCTCATTTCCT	3
H_10	CTTTCTTACCCTCCTCGTTTATCCTACCGCCGTTAGACTTCTGCGCTCT	2
H_11	CTTTCTTACCCTCCTCGTTTATCCTACCACCGTTGGGCTTCTGCGCTCT	1
**H_12**	TCATCCTATCTTCCCTACCCGTTCTGCAGTCGTCAAAGCCCCATTTCCT	7
H_13	CTTTCTTACCCTCCTCGTTTATCCTACAACTGTTAGACTTCTGCGCTCT	1
H_14	CTTTCTTACCCTCCTCGTTTATCCTACCACCGTCAGACTTCTGCGCTCT	1
H_15	CTTTCTTGCCCCCCTCGTTTATCCTACCACCGTTAGACTTCTGCGCTCT	1
H_16	CTTACTTACCCTTCTCGTTTATCCCACCACCGTTAGACTTCTGCGCTCT	1
**H_17**	TCATCCTATCTTCCCTACCCGTTCTGCAGTCGTCAAAGCCCCATTTCCC	1
H_18	CTTTCTCACCCTCCTCGTTTATCCTACAACCGTTAGACTTCTGCGCTCT	1
H_19	CTTTCTTACCCCCCTCGTTTATCCTACCACCATTAGACTTCTGCGCTCT	2
H_20	CTTTCTTACCCTCCTCGTTTATCCTACCGCCGTTAGACTTCTGCGCCCT	1
H_21	CTTACTTACCCTCCTCGTTTATCCTACAACCGTTAGACTTCTGCGCTTT	1
**H_22**	TCATCCTATCCTCACCGCCTGTTCTGTAGTCATCAAACCCTCATTTCCT	1

Table S2. Population pairwise ϕ_{ST} values from *cox1* in octopus' samples.

ϕ_{ST} values, using pairwise comparisons as distance method, are located below diagonal and above diagonal their correspondent p-values (italics). Significance (*p<0.05). Significance after Bonferroni correction (cut off value p<0.0033) in bold.

	**Asturias 2006-2007**	**Asturias 2020-2021**	**Galicia 2020-2021**	**Algarve 2006-2007**	**Algarve 2020-2021**	**Canary Islands 2018-2021**
**Asturias 2006-2007**			*0.25978*	*0.44431*	*0.00861*	*0.35531*
**Asturias 2020-2021**	0.04300		*0.99990*	*0.24017*	*0.17167*	*0.00119*
**Galicia 2020-2021**	0.00933	-0.04895		*0.13226*	*0.24037*	*0.00307*
**Algarve 2006-2007**	0.24908*	0.06832	0.12536		*0.02465*	*0.00020*
**Algarve 2020-2021**	0.00784	0.07626	0.06926	0.15537*		*0.06019*
**Canary Islands 2018-2021**	0.27538*	**0.36418***	**0.35522***	**0.41770***	0.10293	

Table S3. Population pairwise ϕ_{ST} values from CR in octopus' samples.

ϕ_{ST} values, using pairwise comparisons as distance method, are located below diagonal and above diagonal their correspondent p-values (italics). Significance (*p<0.05). Significance after Bonferroni correction (cut off value p<0.0033) in bold.

	**Asturias 2006-2007**	**Asturias 2020-2021**	**Galicia 2020-2021**	**Algarve 2006-2007**	**Algarve 2020-2021**	**Canary Islands 2018-2021**
**Asturias 2006-2007**			*0.25730*	*0.34185*	*0.00822*	*0.45203*
**Asturias 2020-2021**	0.01058		*0.99990*	*0.21077*	*0.11712*	*0.00149*
**Galicia 2020-2021**	0.00270	-0.04978		*0.10187*	*0.10544*	*0.00149*
**Algarve 2006-2007**	0.10147*	0.03509	0.07656		*0.01346*	*0.00020*
**Algarve 2020-2021**	-0.00279	0.09650	0.09608	0.13592*		*0.05396*
**Canary Islands 2018-2021**	0.28215*	**0.38766***	**0.38857***	**0.41558***	0.11231	
